# Ibrutinib-Induced Vasculitis in a Patient with Metastatic Colon Cancer Treated in Combination with Cetuximab

**DOI:** 10.1155/2020/6154213

**Published:** 2020-02-14

**Authors:** Jeffrey Chi, Jennifer Park, Muhammad Wasif Saif

**Affiliations:** Department of Hematology/Oncology, Northwell Cancer Institute, Lake Success, NY, USA

## Abstract

Combination therapy with ibrutinib and cetuximab is being studied in a phase 1b/2 trial in patients with advanced gastrointestinal and genitourinary malignancies. Rash is a common cutaneous adverse effect for both medications. Ibrutinib is a Bruton's tyrosine kinase (BTK) inhibitor approved for the treatment of several hematologic malignancies. The rash can be asymptomatic, nonpalpable, mild skin eruption, or palpable purpuric rash. A rarer panniculitis form has also been reported. Cetuximab, an epidermal growth factor (EGFR) inhibitor, approved for treatment in head and neck and advanced gastrointestinal malignancies is also known to cause acneiform rash in majority of patients. The rash is due to inhibition of EGFR in the basal keratinocytes and hair follicles. In the case of ibrutinib, the off-target effects on EGFR, c-kit, and platelet-derived growth factor receptor (PDGFR) are thought to be responsible for the cutaneous eruption of various forms of rash. The combination therapy with the BTK inhibitor and a direct EGFR inhibitor may potentiate the rash inducing effects of the drugs. Here, we describe a case of vasculitis in a patient with metastatic colon cancer who received both ibrutinib and cetuximab on a phase Ib/II clinical trial.

## 1. Introduction

Combination therapy of ibrutinib and cetuximab is being studied in a phase Ib/II trial in patients with advanced colorectal and genitourinary malignancies who have failed multiple lines of therapy. Cutaneous toxicity is commonly seen in patients when treated individually with ibrutinib or cetuximab. The combined use of ibrutinib and cetuximab may potentiate the cutaneous toxicity.

Ibrutinib, a Bruton's tyrosine kinase (BTK) inhibitor, is approved by Food and Drug Administration (FDA) for the treatment of chronic lymphocytic leukemia (CLL), mantle cell lymphoma (MCL), marginal zone lymphoma (MZL), Waldenstrom macroglobulinemia, and chronic graft versus host disease. Ibrutinib is known to cause rash in 13-27% of CLL and MZL patients [1–4]. The rash can vary from asymptomatic ecchymosis, nonpalpable petechial rash, to leukocytoclastic vasculitis-like palpable purpura and panniculitis [2–4]. The onset of the rash can vary from days to months after initiation of ibrutinib. Mild, nonpalpable rash can be managed with observation without dose disruption, whereas a more severe palpable rash may need topical steroid and require ibrutinib dose disruption. Most patients are able to resume after the resolution of rash.

Cetuximab is an EGFR inhibitor, currently approved by FDA for treatment for head and neck cancer and metastatic colon cancer. One of the most common cutaneous side effects of cetuximab is eruption of acneiform rash over the face and trunk, affecting 45-100% of patients [5]. The rash can range from asymptomatic maculopapular rash to severe generalized exfoliative, ulcerative, or bullous dermatitis [6]. Severity is graded by the Common Terminology Criteria for Adverse Events (CTCAE). Grade 3-4 rash requires dose disruption and treatment with topical antimicrobial cream. The onset of the rash is typically within the first month of the treatment in majority of the patients even though it can occur in any time during the treatment course. Eruption of cutaneous rash has also been shown to have a positive correlation to clinical response of the tumor to cetuximab [7].

Here, we describe a case of vasculitic rash eruption in a patient with metastatic colon cancer who received ibrutinib and cetuximab combination therapy on a phase Ib/II clinical trial.

## 2. Case Presentation

**Figure 1:**
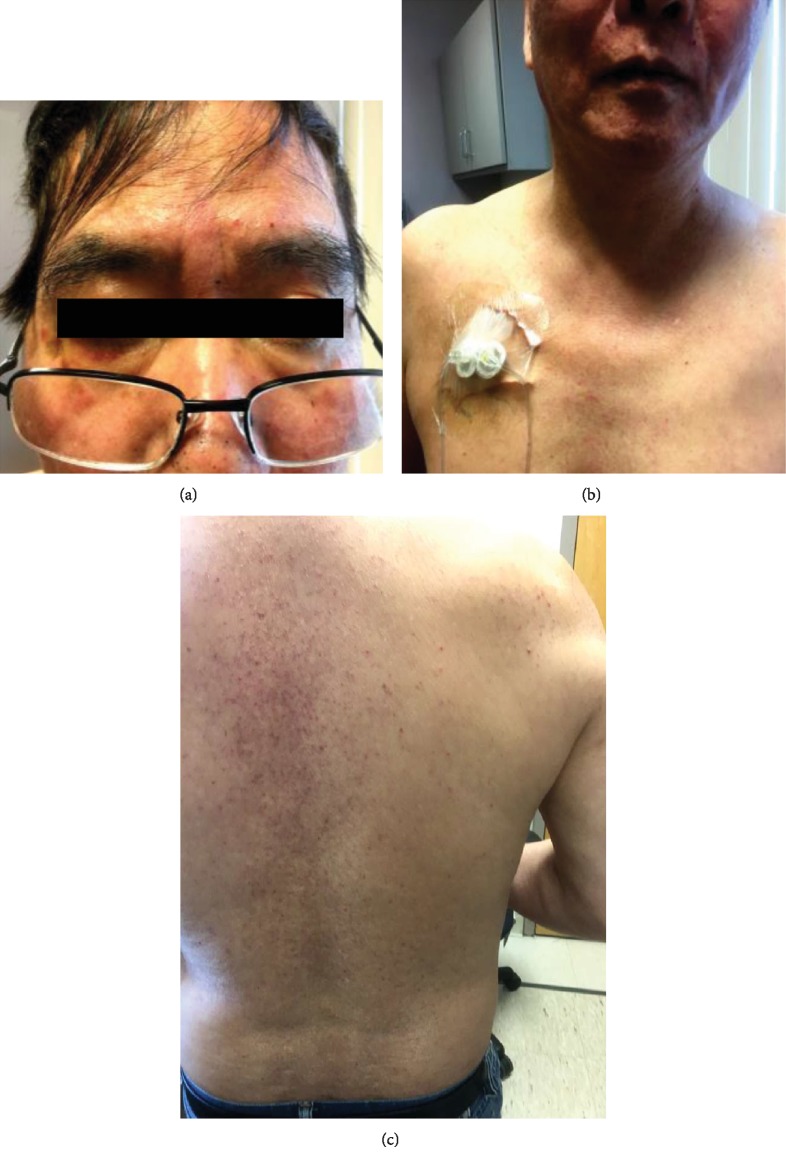
Papular rash with erythematous base: (a) on the face, (b) on the lower face and torso, and (c) on the back.

A 62-year-old male presented with rash one week after he started taking ibrutinib and cetuximab for metastatic colon cancer. The patient history was significant for stage IVa (T4aN2M1a) colon cancer with metastasis to the liver. He underwent partial colon resection and radiofrequency ablation to liver metastasis. The patient received multiple lines of adjuvant chemotherapies (5-fluorouracil (5-FU), leucovorin, oxaliplatin, irinotecan+bevacizumab; 5-FU+bevacizumab; capecitabine+oxaliplatin) from October 2017 to April 2018 due to progression of the disease. He was enrolled into the phase Ib/II study PCYC-1128-CA and received daily ibrutinib and weekly cetuximab combination therapy. One week after the initiation of therapy, the patient presented with macular rash with erythematous base on the face and torso (grade 1). The rash was initially thought to be due to cetuximab which is known to cause acneiform rash that is often associated with pain and pruritus. He was treated with minocycline and clobetasol cream for symptomatic relief. A week later, the rash worsened to involve the arms and back (grade 3) (Figure 1). Cetuximab was held. During that time, he continued to take ibrutinib. However, the rash continued to spread to the lower extremities. Both cetuximab and ibrutinib were held. Within a week, the patient's rash improved to grade 1 on the face and grade 2 on the hands, forearms, and lower extremities. Decision was made to resume cetuximab at half the dose (200 g/m2) and ibrutinib at same dose. However, the rash at the lower extremity worsened significantly. Both agents were held again. Biopsy of the lower extremity rash showed parakeratosis with a subcorneal neutrophilic pustule, perivascular inflammatory infiltrate composed of lymphocytes, neutrophils, and eosinophils in association with leukocytoclasia. Perivascular fibrin deposition was identified. The findings were consistent with small vessel vasculitis. Suspicion for other causes of small vessel vasculitis was low as the rash resolved with discontinuation of both medications. Blood culture, hepatitis B and C panels, and periodic acid-Schiff staining were negative making cryoglobulinemia or infectious associated vasculitis unlikely. Based on the clinical and pathological findings, it was determined that the vasculitic rash was drug induced.

Adverse Drug Reaction Probability Scale (Naranjo) was used to assess the causal relationship of the drugs to the skin rash. It is a questionnaire designed to determine the likelihood of whether an adverse reaction is attributable to the medication. A score of greater than 9 suggests a definite association of the drug to the adverse reaction; a score of 5-8 suggests probable association; a score of 1-4 suggests possible association; and a score of zero suggests an unlikely association of the drug to the adverse reaction. In this case, the score for ibrutinib was 7 and the score of cetuximab was 3, making ibrutinib a more probable cause of the rash. (Table 1).

Subsequently, decision was made to reintroduce ibrutinib at a lower dose and cetuximab at full dose. The patient was able to tolerate the combination therapy without further dose reduction.

## 3. Discussion

The use of ibrutinib has expanded from hematologic malignancies to clinical trials in solid tumors. In our case, it was used in combination with cetuximab in a stage IV colon cancer patient who had progression of disease on multiple lines of treatment. Rash is the most common cutaneous adverse effect of both of these medications. When rash occurred, it was difficult to determine the offending drug.

The cutaneous adverse effects of EGFR inhibitors are believed to be due to inhibition of EGFR in basal keratinocytes and hair follicles [7]. These receptors are highly expressed in skin cells and certain types of tumor cells. Cetuximab-induced rash typically manifests as sterile acneiform, erythematous papules, and pustules that develop on the face, upper trunk, and scalp. It often appears within the first month after starting therapy in 45-100% of the patients. Other EGFR inhibitors (panitumumab, pertuzumab, etc.) and EGFR tyrosine kinase inhibitors (erlotinib, gefitinib, osimertinib, etc.) can also cause a similar form of rash [8]. Histopathology typically shows superficial inflammatory cell infiltrate surrounding upper portion hair follicles or superficial neutrophilic suppurative folliculitis with rupture of epithelia lining. Dermal capillaries, sebaceous glands, and eccrine glands are usually not involved. The eruption of cutaneous rash in patients treated with cetuximab has been shown to positively correlate with tumor response rate in patients with colorectal cancer. Rash can be induced by increasing the dose of cetuximab to improve response rate in patients who do not develop the skin rash at the standard dose [9]. Rash of grade 0-2 per CTCAE can be managed by topical corticosteroids, emollients, and antibiotics. Dose reduction is typically not required. Rash of grade 3 and higher requires dose reduction or cessation.

For ibrutinib, cutaneous adverse events have been reported as one of the most common nonhematologic side effects that occur in 13-27% of patients with hematologic malignancies [2]. The rash can vary from asymptomatic ecchymosis, nonpalpable petechial rash, to leukocytoclastic vasculitis-like palpable purpura and panniculitis [2]. Ecchymosis is due to collagen-dependent platelet activation defect and absent adherence to von Willebrand factor [10]. The eruption of rash is thought to be due to the off-target effect of ibrutinib on EGFR despite its high selectivity of BTK. It has been shown that ibrutinib inhibits EGFR in a dose-dependent manner [11] which in turn can lead to similar cutaneous eruptions seen in patients treated with cetuximab. Another proposed mechanism of ibrutinib-induced rash is through its ability to inhibit platelet-derived growth factor receptor (PDGFR) or c-kit [11]. However, the exact mechanism is unclear. Histopathology shows inflammatory infiltrate with neutrophils, lymphocytes, and eosinophils with associated leukocytoclasia in the superficial dermal layer and perivascular tissues. Lobular and septal panniculitis with associated small vessel vasculitis has also been reported [12]. The onset of the rash can vary from within 7 days to over 1 year after initiation of ibrutinib. The rash is typically grade 0-2 which often resolves spontaneously without any specific treatment. Topical corticosteroid therapy and antihistamine can be given for pruritic rash. Rash of grade 3 and higher requires dose reduction or discontinuation.

Our case is unique because the patient was on combination treatment with ibrutinib and cetuximab. Both of which are known to cause rash. Both drugs, when given individually, inhibit EGFR in a dose-dependent manner. The skin toxicity-inducing effect may be amplified when both medications are used in combination. It is important to discern the causative agent so that appropriate dose adjustment can be made. In our case, ibrutinib, rather than cetuximab, was found to have a higher causal correlation to the rash as assessed by Naranjo's Adverse Drug Reaction Probability Scale. Biopsy of the rash also showed small vessel vasculitis which is more commonly seen in rash associated with ibrutinib but rarely with cetuximab. Dermal capillaries are typically spared in cetuximab rash. The patient was able to tolerate the combination therapy with reduced dose of ibrutinib. Cetuximab was continued at the same dose.

## 4. Conclusion

Diagnosis of ibrutinib-induced vasculitis was challenging in our patient as he was on combination therapy with cetuximab. Clinicians should be aware that despite a higher incidence rate of skin toxicity with cetuximab, ibrutinib can also be the trigger for the rash when the drugs are used in combination. Dose adjustments can be made accordingly once the offending drug is identified.

## Figures and Tables

**Table 1:** Adverse Drug Reaction Probability Scale.

Question	Yes	No	Do not know	Ibrutinib	Cetuximab
1. Are there previous conclusive reports on this reaction?	+1	0	0	1	1
2. Did the adverse event appear after the suspected drug was administered?	+2	-1	0	2	2
3. Did the adverse event improve when the drug was discontinued or a specific antagonist was administered?	+1	0	0	1	0
4. Did the adverse event reappear when the drug was readministered?	+2	-1	0	2	-1
5. Are there alternative causes that could on their own have caused the reaction?	-1	+2	0	-1	-1
6. Did the reaction reappear when a placebo was given?	-1	+1	0	0	0
7. Was the drug detected in blood or other fluids in concentrations known to be toxic?	+1	0	0	0	0
8. Was the reaction more severe when the dose was increased or less severe when the dose was decreased?	+1	0	0	1	1
9. Did the patient have a similar reaction to the same or similar drugs in any previous exposure?	+1	0	0	0	0
10. Was the adverse event confirmed by any objective evidence?	+1	0	0	1	1
			Total	7	3
